# Synergistic effect of copper(ii) ions and orthosilicate group in nanosized hydroxyapatite

**DOI:** 10.1039/d6ra01471b

**Published:** 2026-04-08

**Authors:** Nataliia D. Pinchuk, Agata Piecuch, Paulina Sobierajska, Cole Latvis, Katarzyna Szyszka, Sara Targonska, Oleksii Bezkrovnyi, Rafał Ogórek, Yadong Wang, Rafal J. Wiglusz

**Affiliations:** a Institute of Low Temperature and Structure Research, Polish Academy of Sciences Okolna 2 Wroclaw 50-422 Poland p.sobierajska@intibs.pl r.wiglusz@intibs.pl; b Frantsevich Institute for Problems of Materials Science of the NAS of Ukraine Pritsaka, 3 Kyiv 03142 Ukraine; c Department of Mycology and Genetics, University of Wroclaw Przybyszewskiego 63/77 51-148 Wroclaw Poland; d Meinig School of Biomedical Engineering, College of Engineering, Cornell University Ithaca New York 14853-1801 USA; e Institute of Chemistry, São Paulo State University-UNESP Araraquara São Paulo 14800-060 Brazil

## Abstract

Copper- and silicate-containing hydroxyapatites have attracted increasing attention as potential antimicrobial biomaterials. This paper aims to synthesize nanosized hydroxyapatite-type materials doped with copper(ii) ions alone (nHAp: Cu^2+^) and with orthosilicate groups (Si-nHAp: Cu^2+^) *via* a microwave-assisted hydrothermal method followed by sintering at 450 °C. Copper(ii) ions were incorporated at the concentrations of 0.1, 0.5, and 1 mol%, while orthosilicate substitution replaced one orthophosphate group in the hydroxyapatite structure. This study focuses on the synergistic influence of cationic (Cu^2+^) and anionic (SiO_4_^4−^) substitutions on the composition, morphological features, ion-release behaviour, and biological activity of nanosized apatite materials. We used a wide range of characterization methods and demonstrated both morphological changes of doped hydroxyapatite nanoparticles and selective antimicrobial activity against Gram-positive bacteria. No effects on Gram-negative bacteria or fungi were observed. Silicate ions increase the release of Cu^2+^ ions from 56% to 98%, thereby enhancing the reduction of Gram-positive bacteria growth. At lower concentrations, the materials showed no cytotoxic effect. Our work clearly shows that synthesized materials with low dopant content exhibit selective antimicrobial activity and can be used to treat infections, targeting Gram-positive bacteria such as *S. epidermidis*, *S. aureus*, and *E. faecalis*.

## Abstract 2

Copper- and orthosilicate-doped hydroxyapatites exhibited antibacterial activity, selectively against Gram-positive bacteria, but not against Gram-negative bacteria and fungi, showing the role of silicate groups in enhancing antimicrobial efficacy.

## Graphical abstract

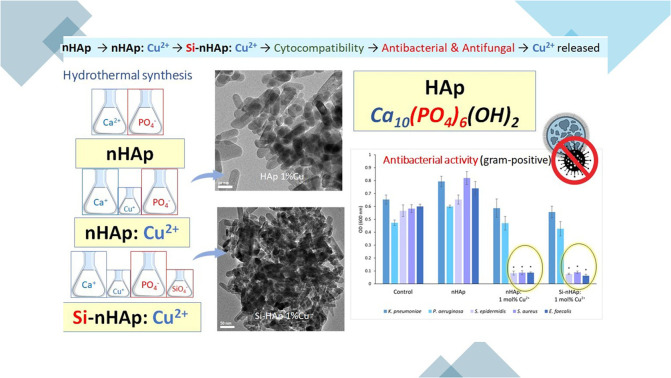

## 1 Introduction

The treatment of musculoskeletal system-related diseases, including afflictions of cartilage and bone tissues, requires the development of innovative bioactive materials for bone tissue replacement. The inorganic mineral component of bone tissue is calcium-deficient hydroxyapatite, which can be recreated synthetically with the stoichiometric formula (Ca_10_(PO_4_)_6_(OH)_2_, abbr. HAp) through various methods.^1^ HAp and its derivatives find application not only in bone and cartilage tissue regeneration but also as carriers for targeted drug delivery and biologically active agents.^1,2^ To develop modern bioactive materials with tailored properties, HAp is often combined with other materials, leading to the creation of composites, or modified through substitution and functionalization.^3^ An essential challenge in bone tissue engineering is the development of biocompatible materials with antibacterial properties.^4^ An element such as copper can become a promising additive to HAp to improve its bioactive, antibacterial, and antifungal properties.^5,6^ The biological activity of copper compounds has therapeutic applications, primarily as antibacterial and anticancer agents. Copper demonstrates a broad range of antimicrobial activity against Gram-positive and Gram-negative bacteria, viruses, and fungi, with its mode of action relying mainly on ROS (reactive oxygen species) generation and membrane disruption.^7^ Various approaches were undertaken to design Cu^2+^ ion-doped hydroxyapatite-type materials with enhanced antimicrobial activity, including HAp loaded with Cu^0^ nanosized particles, Cu^2+^-HAp sintered discs, and coatings, among others.^8–10^ These materials exhibited good antibacterial activity against both Gram-negative and Gram-positive strains. Since research on the biological activity of copper-doped hydroxyapatites has primarily focused on their antibacterial properties, much less is known about their antifungal activity. However, Hassanain *et al.* (2024) showed spore development inhibition in filamentous fungi.^10^ The incorporation of Si groups into the HAp structure may enhance its resorption, which causes faster release of biologically active ions.^11^ However, no data are available on the antimicrobial activity of hydroxyapatites containing SiO_4_^4−^ groups.

In addition to their antimicrobial properties, copper species appear to be effective in addressing inflammatory conditions such as rheumatoid arthritis.^12^ The copper(ii) ion is involved in many biological processes and acts as a modulator of cellular signal transmission.^13^ Moreover, the Cu^2+^ ions are considered multifunctional, participating in a broad spectrum of intracellular processes.^14^ Therefore, to enhance the bioactive properties of hydroxyapatite-type materials, incorporating Cu^2+^ ions into their structure is a promising approach. This can be achieved through various methods, as outlined below. One approach involves the fabrication of Cu^2+^ ion-doped HAp by co-precipitation with Cu(NO_3_)_2_·nH_2_O.^15–19^ Another study investigates scaffolds containing copper ions obtained *via* ion exchange with CuSO_4_ and the 3D extrusion-deposition technique, in which the incorporation of Cu^2+^ ions enhances the scaffolds' antibacterial activity.^20^ Moreover, copper(ii)-silver(i) ion-*co*-doped HAp with enhanced antibacterial activity was synthesized *via* the sol–gel technique combined with ultrasonic irradiation and the addition of Cu(NO_3_)_2_ · 4H_2_O.^21^ Also, Cu^2+^ ion-doped HAp was synthesized by the neutralization method by dissolving CuO in H_3_PO_4_ and adding Ca(OH)_2_ dropwise.^22^ Cu^2+^ ion-doped HAp was also obtained by mixing a washed HAp slurry with Cu(ii) acetate solution.^23^ In another method, Cu^2+^ ion-doped HAp ceramics were prepared by a solid-state reaction, with a mixture of HAp and Cu^2+^ oxide sintered at 1100 °C. During this process, copper with mixed valence replaces hydrogen in the hexagonal channels of the apatite lattice, forming a solid solution.^24^ The Cu^2+^ ion can substitute for Ca^2+^ and Na^+^ ions, occupy vacancies, or enter the hexagonal channels of the apatite lattice. The predominant dissolution–precipitation or coprecipitation mechanism may coexist with adsorption and diffusion mechanisms in calcium-deficient, sodium-containing carbonate hydroxyapatite-type material modified with Cu^2+^ ion.^25^ Solid-state synthesis of stoichiometric HAp with copper oxide results in the formation of pure copper-doped HAp phases up to *x*Cu^2+^ ≤0.7 in the theoretical formula Ca_10_Cu_*x*_(PO_4_)_6_O_*y*_H_*z*_. In contrast, coprecipitation using calcium and copper nitrates yields stable copper-doped HAp phases after low-temperature calcination (≤600 °C), where Cu^2+^ ions primarily substitute calcium(ii) ions at the Ca(2) site, following the formula.^26^ These differences highlight the significant influence of the synthesis method on the incorporation of Cu^2+^ ions, affecting both its substitution site and valence state. A second important factor in our research is the incorporation of silicon, an essential element for the regeneration of mammalian tissues. The biological role of silicon in health, especially in bone health, remains unclear, although it is suggested to play a role in collagen synthesis and matrix mineralization.^27^ Orthosilicate groups can be introduced into the hydroxyapatite-type structure by replacing orthophosphate groups.^28–31^ Studies have shown that the appropriate incorporation of orthosilicate groups into nanosized calcium phosphate scaffolds significantly enhances osteoblast adhesion and proliferation *in vitro*.^29^ Silicate-substituted HAp (abbr. Si-HAp) can be synthesized using various methods, including precipitation^30,31^ and hydrothermal methods.^29,32^ Furthermore, surgical site infections (SSIs) pose a significant public health challenge, contributing to increased morbidity, mortality, and healthcare costs worldwide. Trauma patients, especially those undergoing emergency surgery or those with open fractures, are at increased risk of SSIs due to the severity of their injuries and increased exposure to pathogens. These findings underscore the importance of preventive measures, such as appropriate preoperative interventions, strict adherence to aseptic technique, and appropriate prophylaxis, to reduce the incidence of SSIs and improve patient outcomes. These infections are typically hospital-acquired. In light of our considerations, apatite-type materials appear to be ideal candidates for such studies, as they can exhibit potent antibacterial properties.^33^

It is also possible to obtain Si-HAp with varying degrees of substitution of PO_4_^3−^ groups by SiO_4_^4−^ groups.^34^ This substitution induces changes in the crystal lattice of the apatite-type structure. Therefore, the production of Si-HAp doped with Cu^2+^ ions may be promising, as this material could combine and strengthen the biologically active properties of both orthosilicate groups and Cu^2+^ ions. For instance, we successfully synthesized nanosized Si-HAp materials with various cationic substitutions, including europium(iii) and strontium(ii) ions, *via* hydrothermal microwave synthesis.^32^ However, serious side effects can occur when intracellular free copper exceeds the normal threshold.^4,35^ Therefore, it is essential not to introduce excessive amounts of Cu^2+^ ions into any medical supplies. Most papers on Cu^2+^ ion-doped hydroxyapatite-type materials involve doping levels above 1 mol%, so it is crucial to develop and study materials with low Cu^2+^ ion content.^4,35^ In our work, we chose nHAp and Si-nHAp, and a low concentration of the Cu^2+^ ion (≤1 mol% relative to Ca^2+^) for doping.

The presented work aimed to obtain nanosized hydroxyapatite-type materials enriched with Cu^2+^ ions and orthosilicate groups, meeting the challenging task of combining high biocompatibility with antibacterial and antifungal activities.

## 2 Materials and methods

### 2.1 Materials

#### 2.1.1 Starting substrates

Calcium nitrate tetrahydrate Ca(NO_3_)_2_·4H_2_O (99.0–103.0%, Alfa Aesar), copper(ii) nitrate hemi(pentahydrate) Cu(NO_3_)_2_·2.5H_2_O (98.0–102.0%, Alfa Aesar), diammonium hydrogen phosphate (NH_4_)_2_HPO_4_ (>99.0%, Acros Organics), and tetraethyl orthosilicate TEOS (>99%, Alfa Aesar) were used for the synthesis of doped hydroxyapatites. Ammonia solution (NH_3_·H_2_O, 25%, Avantor) was used to control the pH level.

#### 2.1.2 Synthesis of the nanosized hydroxyapatite (nHAp)

Nanosized hydroxyapatite (nHAp) was synthesized *via* a microwave-assisted hydrothermal method based on previously reported procedures, as described in the previous papers.^32,36^ The amounts of the initial components were calculated for the synthesis of 2 g of hydroxyapatite with a stochiometric Ca/P molar ratio of 1.67. Two precursor solutions were prepared separately by dissolving the appropriate stochiometric quantities of the reagents in 30 mL of deionized water: a cation source solution containing Ca(NO_3_)_2_·4H_2_O as a source of Ca^2+^ ions and an anion source solution containing (NH_4_)_2_HPO_4_ as a source of PO_4_^3−^ ions. The anion and cation source solutions were mixed in a Teflon vessel, with the pH maintained at 9–10 by ammonia solution. The hydrothermal synthesis was carried out in a microwave reactor (ERTEC MV 02-02, Poland) at 240–250 °С and 42–45 bar for 90 min. Synthesized material was dried at 80 °C and sintered at 450 °C. The prepared material was labeled as nHAp.

#### 2.1.3 Synthesis of the copper(ii)-doped nanosized hydroxyapatite (nHAp: Cu^2+^)

Copper(ii)-doped nanosized hydroxyapatite (nHAp: Cu^2+^) was synthesized using a hydrothermal method as described in the previous papers^32,36^ and a procedure analogous to that described in Section 2.1.2. Two precursor solutions were prepared: a cation source solution containing Ca(NO_3_)_2_·4H_2_O and Cu(NO_3_)_2_·2.5H_2_O as a source of Ca^2+^ and Cu^2+^ ions, respectively, and an anion source solution containing (NH_4_)_2_HPO_4_ as a source of PO_4_^3−^ ions. The amount of copper precursor was adjusted to prepare 0.1, 0.5, and 1 mol% of the Cu^2+^ ions relative to the molar content of Ca^2+^ ions, while maintaining the overall stoichiometry of the hydroxyapatite structure. Mixing of the solutions, hydrothermal synthesis, drying, and sintering were carried out under the same conditions as described for nHAp. The prepared materials were labeled as nHAp: 0.1 mol% Cu^2+^, nHAp: 0.5 mol% Cu^2+^, and nHAp: 1 mol% Cu^2+^.

#### 2.1.4 Synthesis of the copper(ii)-doped nanosized orthosilicate-substituted nanosized hydroxyapatite (Si-nHAp: Cu^2+^)

Copper(ii)-doped orthosilicate-substituted nanosized hydroxyapatite (Si-nHAp: Cu^2+^) was synthesized using a hydrothermal method as described in the previous papers^32,36^ and a procedure analogous to that described in Section 2.1.2. Two precursor solutions were prepared: a cation source solution containing Ca(NO_3_)_2_·4H_2_O and Cu(NO_3_)_2_·2.5H_2_O as a source of Ca^2+^ and Cu^2+^ ions, respectively, and an anion source solution ((NH_4_)_2_HPO_4_) as a source of PO_4_^3−^ groups. Also, tetraethyl orthosilicate (TEOS) was used as a source of the SiO_4_^4−^ group. The amount of copper precursor was set to prepare 0.1, 0.5, and 1 mol% of Cu^2+^ ions relative to the molar content of Ca^2+^ ions, and the amount of TEOS was calculated to introduce one orthosilicate group per apatite formula unit (replacing one PO_4_^3−^ group with one SiO_4_^4−^ group). The anion substrates were added to the cation source solution in the following sequence: firstly, TEOS, then (NH_4_)_2_HPO_4_ solution. Hydrothermal synthesis, drying, and sintering were carried out under the same conditions as described for nHAp. The obtained materials were labeled as Si-nHAp: 0.1 mol% Cu^2+^, Si-nHAp: 0.5 mol% Cu^2+^, and Si-nHAp: 1 mol% Cu^2+^.

### 2.2 Methods

#### 2.2.1 X-ray powder diffraction (XRPD)

The structural and crystalline characteristics of the synthesized materials were analyzed using a PANalytical X'Pert Pro X-ray diffractometer (Malvern Panalytical Ltd, Malvern, UK). The measurements were conducted using Cu–Kα radiation over a 2*θ* range of 10 °–80°, with an acquisition time of 2 hours. The obtained diffraction patterns were compared with reference standards from the Inorganic Crystal Structure Database (ICSD), specifically hydroxyapatite (ICSD-151941) and orthosilicate-substituted hydroxyapatite (ICSD-32357).

#### 2.2.2 Energy-dispersive X-ray spectroscopy (EDS)

The chemical composition of the synthesized materials was analyzed using a field-emission scanning electron microscope (FE-SEM, FEI Nova NanoSEM 230) equipped with an energy-dispersive X-ray spectrometer (EDS, EDAX Apollo X Silicon Drift Detector) operated with Genesis EDAX Microanalysis Software. For measurements, the samples were embedded in carbon resin (PolyFast Struers) and pressed using an automatic press (CitoPress-1, Struers). EDS analyses were performed at an accelerating voltage of 30.0 kV, targeting a large analysis area of 50 × 200 µm. For each sample, EDS spectra were recorded in triplicate, and the reported values represent the mean of these measurements. EDS analysis was used to measure the concentrations of Ca, P, O, Si, and Cu in the obtained materials.

#### 2.2.3 High-resolution transmission electron microscopy (HRTEM)

The morphology of prepared samples was determined by high-resolution transmission electron microscopy, using a Philips CM-20 SuperTwin instrument operating at 160 kV. Specimens were prepared by dispersing the sample in ethanol and placing a droplet of the suspension on a carbon-coated copper grid. Samples were then dried and purified using an oxygen/hydrogen plasma cleaner. Statistical analysis of the TEM measurement data was conducted, including the construction of particle width and length distribution diagrams, along with a grain size distribution curve.

#### 2.2.4 ICP-OES elemental assay

For the ICP-OES measurements, the samples were digested in diluted suprapur nitric acid (Merck) using the ERTEC MV 02-02 microwave reactor (Poland). The results were presented as the mean concentration value (*n* = 3) in ppm (mg L^−1^), along with the measurement precision expressed as %RSD. The emission line intensities of Ca, Cu, P, and Si elements were measured using a PerkinElmer AVIO 220 inductively coupled plasma optical emission spectrometer (ICP-OES). The following analytical lines were selected for the measurements: 315.89 nm (Ca), 327.39 nm (Cu), 213.62 nm (P), and 251.61 nm (Si). Calibration was performed using standard solutions in the concentration range of 0.2–5.0 mg L^−1^. Analytical signals for all elements were measured in triplicate (*n* = 3) using previously diluted sample solutions: 100× for Ca and P, 5× for Cu and Si.

#### 2.2.5 Fourier-transformed infrared spectroscopy (FT-IR)

FT-IR spectroscopy was employed to identify characteristic molecular vibrations. Spectra were recorded using a Thermo Scientific Nicolet iS50 FT-IR spectrometer, equipped with an Automated Beamsplitter Exchange system (iS50 ABX with a DLaTGS KBr detector), an integrated all-reflective diamond ATR module (iS50 ATR), and a Thermo Scientific Polaris™ system. A HeNe laser served as the infrared radiation source. The FT-IR spectra were acquired using the ATR module, covering the mid-infrared range.

#### 2.2.6. Antibacterial and antifungal activity

Antibacterial and antifungal activity of Cu^2+^ ion-doped nanosized hydroxyapatite-type materials was tested against the following bacteria: *Staphylococcus aureus* ATCC 6538, *Staphylococcus epidermidis* ATCC 12228, *Enterococcus faecalis* ATCC 51299, and *Klebsiella pneumoniae* subsp.*pneumoniae* ATCC 700603, *Pseudomonas aeruginosa* ATCC 27853, and fungi: *Candida albicans* ATCC 90028, Candida kruzei ATCC 2159, and *Candida tropicalis* ATCC 13803 strains as previously described.^36,37^ Nanosized apatite-type material dispersions (10 mg mL^−1^) were prepared, sonicated, and diluted 10× in minimal medium. For antibacterial testing, 96-well plates were filled with bacterial cultures (0.5 McF, 10× diluted), nanosized apatite solutions, and medium, incubated at 37 °C for 18 h, and then the OD600 was measured. Antifungal testing followed a similar protocol in SD medium.

#### 2.2.7 Cytocompatibility evaluation

The cytocompatibility testing of Cu^2+^ ion-doped nanosized hydroxyapatite-type materials against human umbilical vein endothelial cells (HUVECs) was performed in accordance with ISO:10993-5. All nanoparticles were sterilized *via* autoclaving (121 °C) and handled aseptically. HUVECs (passage 5) were grown in three 96-cell TCPS plates with endothelial growth medium supplemented with 5% fetal bovine serum, growth factors, ascorbic acid, and hydrocortisone (Promocell C-22121). Once confluency was reached, the media was replaced with media containing nanoparticles at 0.01, 0.1, 1 mg mL^−1^, or fresh media. After 24, 48, and 72 hours, one plate was removed from the incubator. The nanoparticle-containing media were replaced with fresh media, and the cells were immediately treated with CellTiter-Glo® 2.0 Cell Viability Assay (Promega) in accordance with the manufacturer's instructions. Luminescence measurements were recorded on a microplate reader. Background luminescence from assay reagent mixed into sterile media was recorded each day and subtracted from the sample readings.

#### 2.2.8 ICP-MS quantification of released ions

ICP-MS evaluation of Cu^2+^ ion release was performed using Cu^2+^ ion-doped nHAp and Si-nHAp. Each particle type was suspended in supplemented serum-containing cell media (Promocell C-22121) at 1000 µg mL^−1^ and incubated at 37 °C on a shaker. After 24, 48, and 72 hours, samples were taken and filtered through a 0.2 µm syringe filter to remove nanoparticle aggregates. The samples were then digested in 2% trace-metal-grade nitric acid (Oakwood Chemical) to a final volume of 10 mL. Samples were analyzed by an Agilent 7800 ICP-MS for Cu^2+^ ion concentrations (*m*/*z*: Cu, 63) using a mix standard (BDH82026-108, Avantor, VWR Funding Inc., Radnor, Pennsylvania, United States) and an ICP-MS internal standard (5188-6525, Allegiant Technology, Granada Ln, Overland Park, KS 66211, United States). Quality control was performed by periodically measuring standards, blanks, and replicate samples.

## 3 Results and discussion

### 3.1 X-ray powder diffraction analysis

**Fig. 1:**
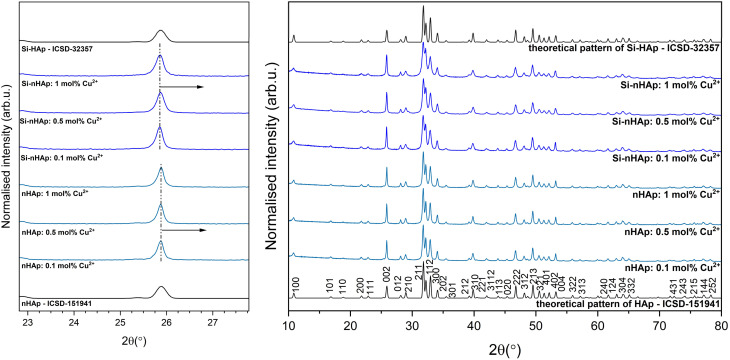
XRPD patterns of hydroxyapatite nanoparticles doped with copper in the amount of 0.1, 0.5, and 1 mol% (labeled as nHAp: 0.1 mol% Cu^2+^, nHAp: 0.5 mol% Cu^2+^ and nHAp: 1 mol% Cu^2+^) and orthosilicate-substituted hydroxyapatite nanoparticles doped with copper in the amount of 0.1, 0.5 and 1 mol% (labeled as Si-nHAp: 0.1 mol% Cu^2+^, Si-nHAp: 0.5 mol% Cu^2+^ and Si-nHAp: 1 mol% Cu^2+^), prepared *via* a hydrothermal method, followed by sintering at 450 °C. ICSD patterns of pure hydroxyapatite (HAp) and orthosilicate-substituted hydroxyapatite (Si-HAp) are presented.

nHAp and Si-nHAp doped with 0.1, 0.5, or 1 mol% of Cu^2+^ ions were assessed by X-ray powder diffraction (XPD). The analysis showed the presence of the hydroxyapatite crystal phase and the absence of other phases for all obtained nanosized materials. Fig. 1 shows these diffractograms compared with pure hydroxyapatite (ICSD-151941) and orthosilicate-substituted hydroxyapatite structures (ICSD-32357) from the Inorganic Crystal Structure Database.^38,39^ The absence of additional diffraction peaks attributable to secondary phases confirms the formation of single-phase materials, with no evidence of other silica-based compounds. For all materials, we observed diffraction peaks at 25.9°, 31.8°, 32.2°, and 32.9° corresponding to the (002), (211), (112), and (300) crystallographic planes, respectively, which are characteristic of the hexagonal hydroxyapatite structure of pure HAp (ICSD-151941). No additional peaks originating from other phases were observed. This is similar to the findings in,^18^ where the replacement of Ca^2+^ by Cu^2+^ ions in the crystal structure was demonstrated for Cu^2+^ ion-doped nanosized hydroxyapatite-type materials prepared by a wet-chemistry method. The absence of secondary copper-containing phases indicates that Cu^2+^ ions are incorporated into the apatite structure rather than forming separate crystalline phases. The substitution of Ca^2+^ ions by Cu^2+^ ions in the crystal structure was also confirmed by the shift in the diffraction peak positions (Fig. 1, left). Hydroxyapatite, and especially Si-HAp doped with Cu^2+^ ions, shows a shift in the position of the (002) plane (c plane) towards higher 2*θ* angles.^18^ This is associated with a decrease in cell parameters due to the substitution of the larger Ca^2+^ cation (CN9 = 1.18 Å, Ca^2+^ CN7 = 1.06 Å, where CN is the coordination number) by the smaller Cu^2+^ cation (CN7 = 0.73 Å).

The obtained results are consistent with those reported in,^22^ where XRD patterns of Cu^2+^ ion-doped hydroxyapatite-type materials showed that samples prepared by a modified neutralization method in an inert atmosphere (N_2_) exhibited a pure apatite phase, with sharp peaks confirming their high crystallinity. Additionally, the purity of the Cu^2+^ ion-doped HAp samples prepared by co-precipitation and annealed at lower temperatures (*T* ≤ 600 °C) was confirmed through XRD analysis.^26^

### 3.2 Elemental analysis

**Fig. 2:**
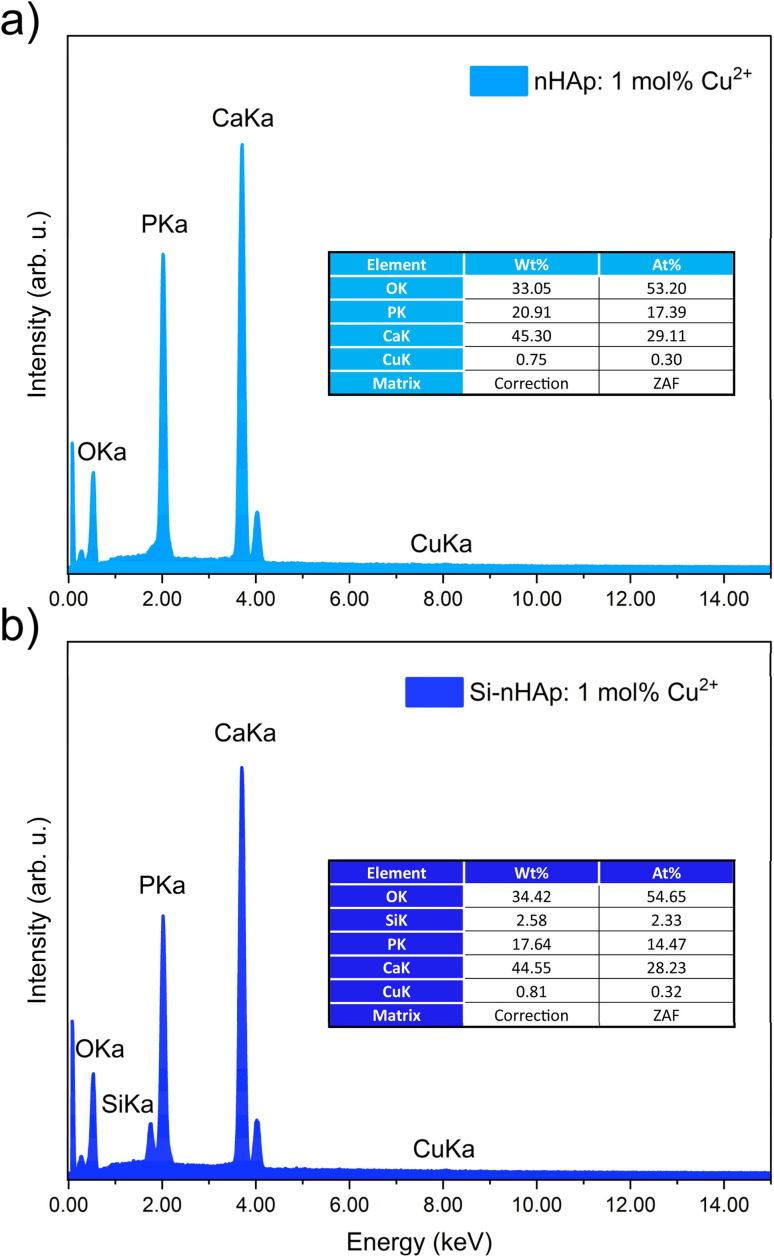
EDS analysis of the 1 mol% Cu^2+^ ion-doped nHAp (a) and 1 mol% Cu^2+^ ion-doped Si-nHAp (b).

The EDS analysis confirmed the presence of calcium, phosphorus, copper, and oxygen elements in the expected quantities for the obtained nHAp materials, as shown in Table 1. For the Cu^2+^ ion-doped Si-nHAp, the analysis additionally detected the presence of silicon along with the other elements. Fig. 2 presents the EDS results for the nHAp: 1 mol% Cu^2+^ and Si-nHAp: 1 mol% Cu^2+^ nanosized hydroxyapatites. The ratio of the elements in both Cu^2+^ ion-doped HAp and Cu^2+^ ion-doped Si-HAp exhibits slight deviations from the ideal stoichiometric hydroxyapatite ratio of 1.67 within the measurement error.

**Table 1:** Elemental content of synthesized nanosized hydroxyapatites based on the EDS analysis

Nanosized apatite-type material	Element content^a^								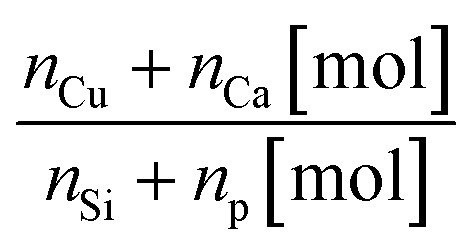	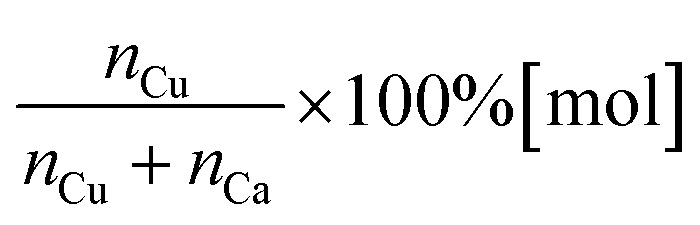
	Cu [at%]	*n*_Cu_	Ca [at%]	*n*_Ca_	Si [at%]	*n*_Si_	P [at%]	*n*_P_		
nHAp	—	—	28.85	10.48		—	17.06	6.00	1.69	—
nHAp: 1 mol% Cu^2+^	0.30	0.1031	29.11	10.05	—	—	17.39	6.00	1.69	1.016
Si-nHAp: 1 mol% Cu^2+^	0.21	0.1129	27.28	10.09	1.99	0.83	15.68	5.17	1.70	1.106

a The relative errors of the EDS method are less than 2%, 4% and 50% for main (above 20 at%), major (20–5. at%) and trace (1–0.1) elements, respectively.

For nHAp doped with 1 mol% Cu^2+^ ions, a slight increase in the (*n*_Ca_ +*n*_Cu_)/(*n*_P_) ratio to 1.69 was observed, which is similar to the stoichiometric value of 1.67. This trend aligns with previous findings^40^ where pure HAp synthesized *via* the aqueous precipitation method and sintered at 1100 °C also showed a Ca/P ratio of 1.7.

For the Si-nHAp doped with 1 mol% Cu^2+^ ion sample, the (Ca + Cu)/(Si + P) ratio is 1.70. Literature data demonstrated that Si-substituted hydroxyapatite materials synthesized using two different precipitation routes have a Ca/(P + Si) ratio of 1.694 with calcium nitrate and diammonium hydrogen phosphate, whereas with calcium hydroxide and phosphoric acid, this ratio was 1.804.^41^

ICP-OES measurements were performed to determine the total ion concentration in the tested materials. The results are summarized in Table 2. The total Cu^2+^ ion content was 0.85 mol%, which is close to 1 mol%. The silicon content was 12.8 mol%, corresponding to 0.77 mol of Si out of the total Si and P content (6 mol). This value is close to 1, indicating that the material contains one SiO_4_^4−^ group. This value was predicted based on the synthesis. All these results are more reliable than EDS data, because it analyzes the entire sample volume, whereas EDS only examines the surface. Nevertheless, both methods complement each other when making conclusions.

**Table 2:** ICP-OES elemental assay of synthesized nanosized apatite compounds

Material	Ca [mg L^−1^][RSD, %]	Cu [mg L^−1^] [RSD, %]	P, mg L^−1^ [RSD, %]	Si, mg L^−1^ [RSD, %]	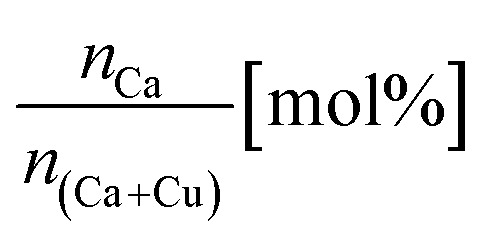	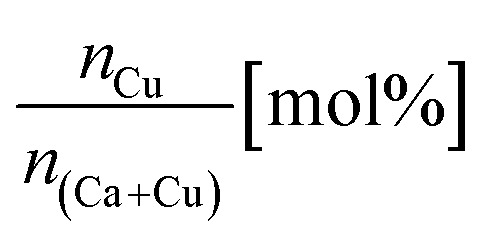	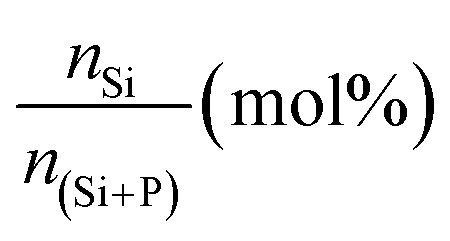	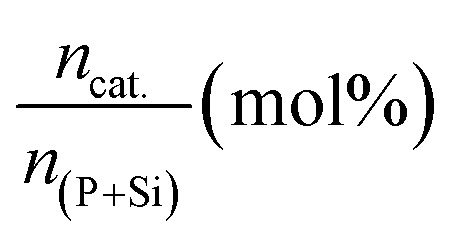
nHAp	394.6 (1.0)	—	162.9 (0.5)	—	100	—	—	1.86
nHAp: 1 mol% Cu^2+^	392.2 (0.2)	5.34 (0.3)	163.5 (0.3)	—	99.15	0.85	—	1.87
Si-nHAp: 1 mol% Cu^2+^	375.4 (0.6)	5.09 (0.8)	130.6 (0.3)	17.3 (0.2)	99.15	0.85	12.8	1.95

### 3.3 FT-IR analysis

**Fig. 3:**
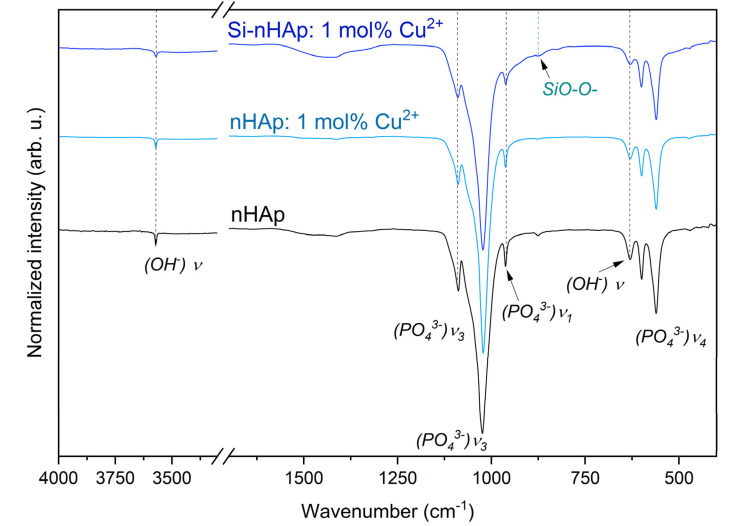
IR spectra of the pure nHAp,^36^ nHAp: 1 mol% Cu^2+,^ and Si-nHAp: 1 mol% Cu^2+^.

Analysis of the infrared spectra of the obtained materials confirmed the presence of functional groups characteristic of hydroxyapatite (Fig. 3). It is well known that vibrations of the phosphate groups (PO_4_^3−^ tetrahedron) originate correspond to four different frequencies: *ν*_1_ – symmetric P–O stretching (938 cm^−1^), *ν*_2_ – symmetric O–P–O bending (420 cm^−1^), *ν*_3_ – asymmetric P–O stretching, P motion (1017 and 1040 cm^−1^), and *ν*_4_ – asymmetric O–P–O bending (567 cm^−1^).^42^ These characteristic modes were recorded for all obtained materials. For pure nHAp, vibration of hydroxyl groups (OH^−^), namely the asymmetric O–H stretching, is a silent mode and occurs at around 3572 cm^−1^, which corresponds to around 3575 cm^−1^ in computed *ab initio* predictions using a periodic approach in.^42^ The same band was observed in the infrared spectrum of the nHAp: 1 mol% Cu^2+^ sample, but with lower intensity at 3571 cm^−1^. In the Si-nHAp: 1 mol% Cu^2+^, the OH^−^ group vibration appeared at 3573 cm^−1^ and exhibited the lowest intensity compared to pure nHAp and nHAp: 1 mol% Cu^2+^. The intensity of hydroxyl groups vibration at 3575 cm^−1^ follows this order: nHAp > nHAp: 1 mol% Cu^2+^ > Si-nHAp: 1 mol% Cu^2+^.

The presence of orthosilicate groups in the synthesized nanosized material is confirmed by the presence of a band at 870 cm^−1^ assigned to the Si–O vibration modes of SiO_4_^4−^ groups. Additionally, a weak absorption line at 450 cm^−1^ is observed, assigned to the Si–O rocking mode. The expected strong Si–O–Si stretching vibration is overlapped by the P–O asymmetric stretching bond.^43^

### 3.4 High-resolution transmission electron microscopy

**Fig. 4:**
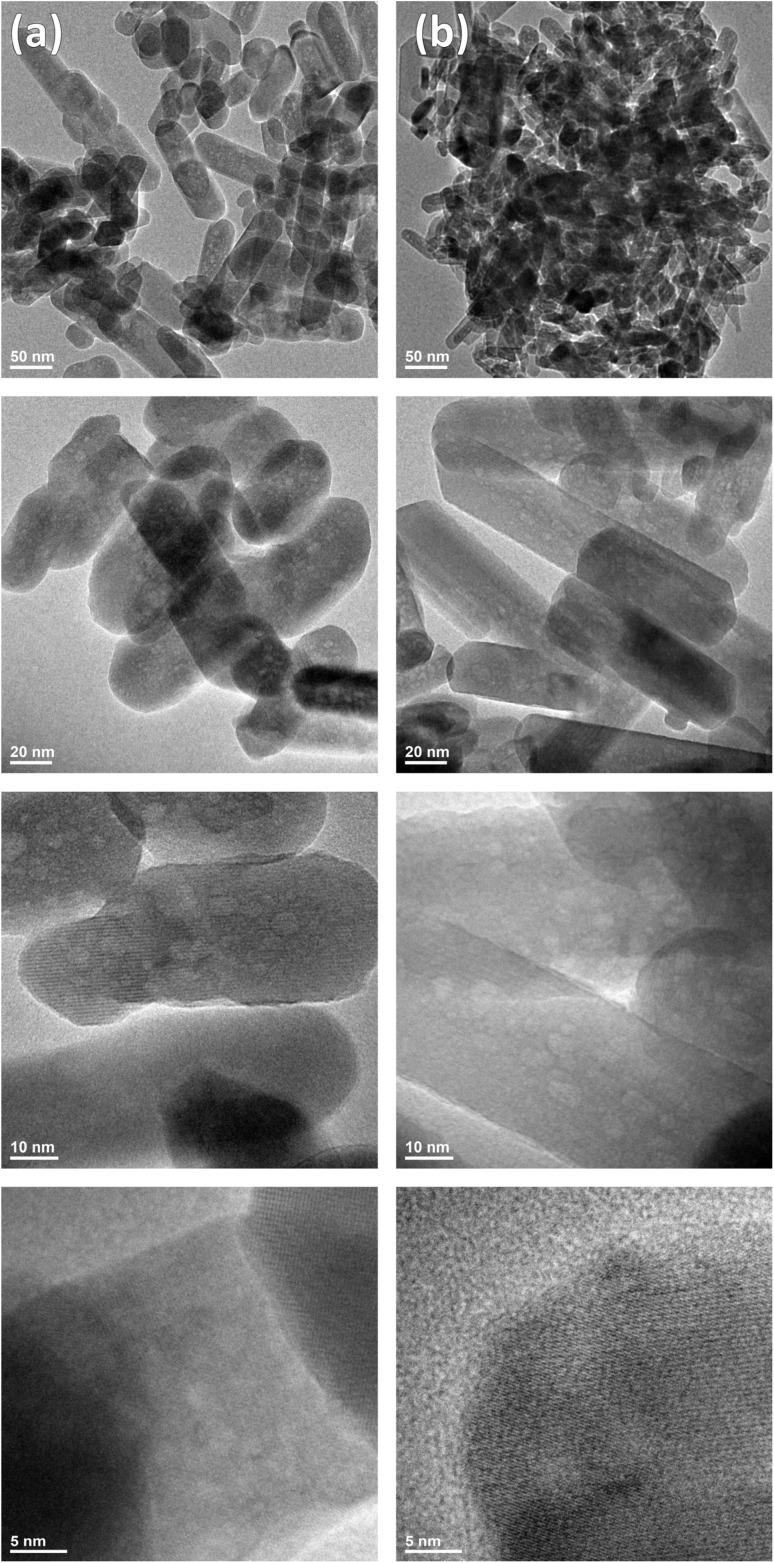
TEM images of nHAp: 1 mol% Cu^2+^ (a) and of Si-nHAp: 1 mol% Cu^2+^ (b) nanosized particles.

High-resolution transmission electron microscopy also confirmed that the prepared nHAp doped with 1 mol% Cu^2+^ ions and Si-nHAp doped with 1 mol% Cu^2+^ ions are nanosized materials. The morphology of these materials is presented in Fig. 4. In general, the materials obtained exhibit nanorod structures. However, nHAp: 1 mol% Cu^2+^ nanoparticles appear more elongated. In comparison to pure nHAp, the crystal size decreases upon Cu^2+^ ion-doping and even more so with SiO_4_^4−^ co-doping. In pure nHAp, the average particle size is 96 nm in length and 48 nm in width, as reported in our previous study.^36^ For nHAp: 1 mol% Cu^2+^, the average particle size reduces to 76 nm in length and 33 nm in width, indicating that doping with 1 mol% Cu^2+^ ion decreases the average particle size in both dimensions. Furthermore, for Si-nHAp: 1 mol% Cu^2+^, the average particle size further decreases to 64 nm in length and 26 nm in width. These results correlate with findings from a study,^44^ which reported that Si-nHAp crystallite size decreases with increasing orthosilicate group content.

**Fig. 5:**
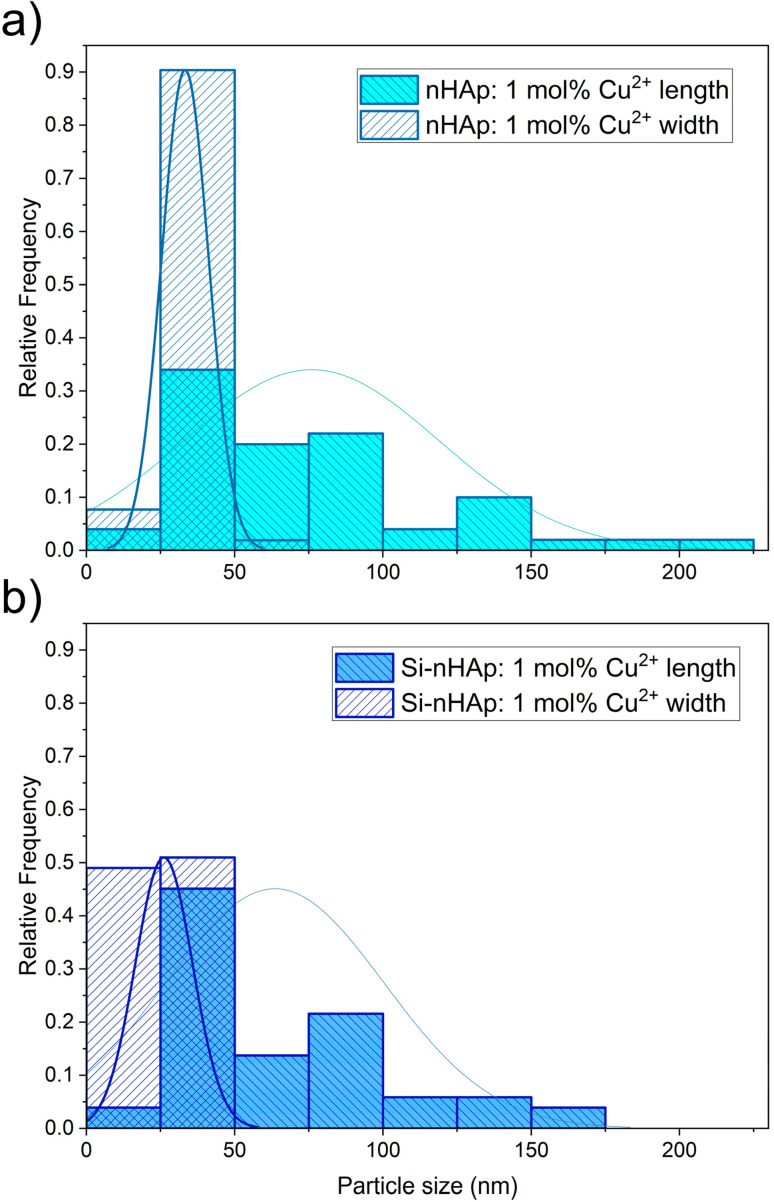
Particle size distribution of the nHAp: 1 mol% Cu^2+^ (a) and of Si-nHAp: 1 mol% Cu^2+^ (b) nanosized materials based on TEM images.

The particle size distribution of the obtained materials is shown in Fig. 5. These histograms show the maximum values of the particle-size distributions, which vary across the prepared materials. The particle size distribution of pure nHAp shows an average peak with a main fraction of 25–50 nm in length and a small peak with an average fraction of 20–40 nm in width, as described in our previous work.^36^ Doping with 1 mol% Cu^2+^ ions increases the average particle size to 25–50 nm, with a pronounced maximum in the particle distribution for length and a smaller maximum in the particle distribution for width. The substitution of one phosphate group with the orthosilicate group in Si-nHAp doped with 1 mol% Cu^2+^ ions leads to decreases of almost 50% of the average fraction 25–50 nm for length, with the formation of a similar maximum of particle distribution with a fraction less than 25 nm and saving a small maximum of distribution with the average fraction 25–50 nm for width.

### 3.5 Antimicrobial activity of hydroxyapatite nanoparticles

**Fig. 6:**
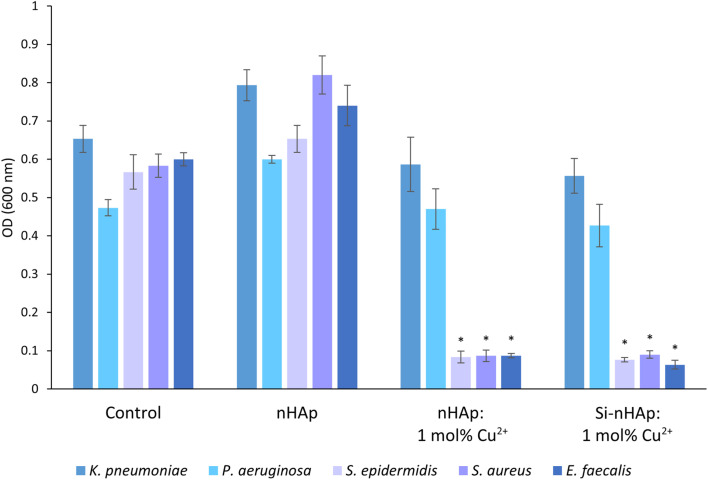
The inhibition of bacterial (*Klebsiella pneumoniae*, *Pseudomonas aeruginosa*, *Staphylococcus epidermidis*, *Staphylococcus aureus*, and *Enterococcus faecalis*) growth by HAp,^36^ nHAp: 1 mol% Cu^2+^, and Si-nHAp: 1 mol% Cu^2+^ (concentration of 1 mg mL^−1^). Bacteria grown without hydroxyapatite-type materials were the control group. Mean ± SD; *n* = 3; *p < 0.05.

**Fig. 7:**
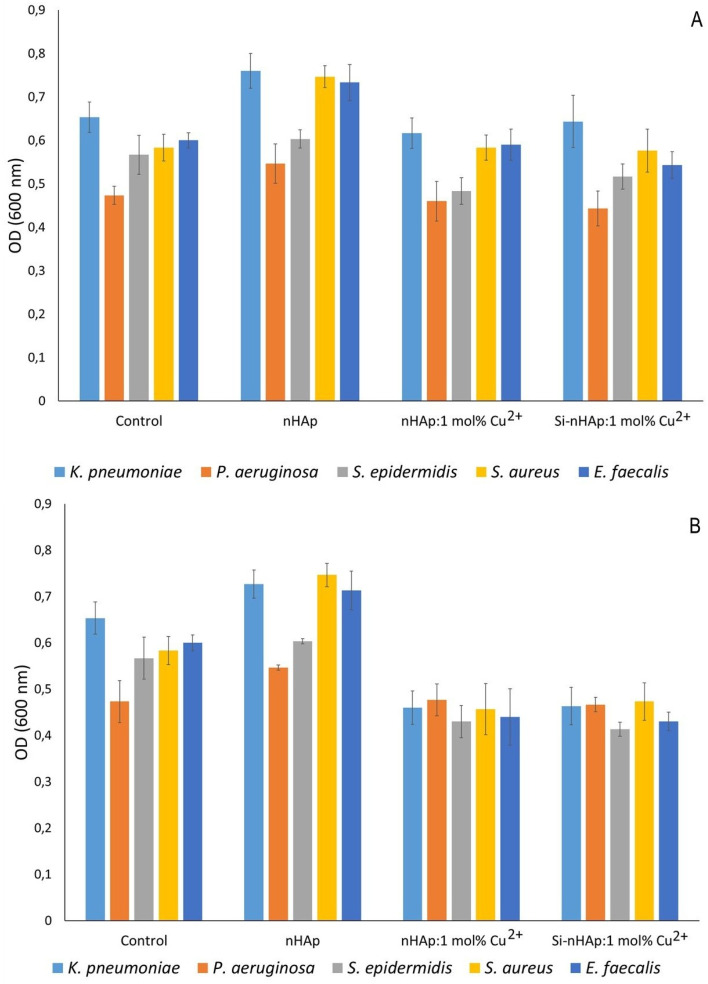
The effect of nHAp,^36^ nHAp: 1 mol% Cu^2+^ and Si-nHAp: 1 mol% Cu^2+^ (A – 0.01 mg mL^−1^; B – 0.1 mg mL^−1^) on bacterial (*Klebsiella pneumoniae*, *Pseudomonas aeruginosa*, *Staphylococcus epidermidis*, *Staphylococcus aureus*, and *Enterococcus faecalis*) growth. Bacteria grown without hydroxyapatite materials were the control group. Mean ± SD; *n* = 3; *p < 0.05.

The inhibitory effect of Cu^2+^ ion-doped hydroxyapatites against Gram-positive (*S. epidermidis*, *S. aureus*, *E. faecalis*) and Gram-negative (*K. pneumoniae* and *P. aeruginosa*) bacteria is shown in Fig. 6. Significant growth reduction was observed for both tested Cu^2+^ ion-doped nHAps (silica-modified and unmodified), but only against Gram-positive species at 1 mg mL^−1^, with no effect at lower concentrations (Fig. 7). The antimicrobial activity of copper is well documented.^45–47^ Its mode of action involves disruption of the bacterial membrane potential, thereby increasing membrane permeability and disrupting cellular homeostasis. Additionally, copper ions induce intracellular production of reactive oxygen species (ROS), leading to oxidative stress and damage to essential cellular components. This oxidative stress results in DNA damage, including strand breaks and impaired replication, as well as lipid and protein oxidation, ultimately leading to enzyme inactivation, structural destabilization, and bacterial cell death.^46^ The disparity in activity against these two groups of bacteria might be attributable to the differences in cell envelope composition. Gram-negative bacteria possess an outer membrane with LPS (lipopolysaccharides), which makes them less susceptible to antimicrobial compounds. Additionally, some species, such as *K. pneumoniae*, can produce capsules. Such structures hinder the penetration of antimicrobial compounds into the cell. On the other hand, the thick cell wall of Gram-positive bacteria may also constitute a barrier to the penetration of substances.^48^ The impact of these differing protective strategies on the selectivity of antibacterial agents is reflected in the literature for hydroxyapatite-type materials doped with Cu^2+^ ions. Noori *et al.* (2024) compared growth inhibition by Cu^2+^ ion doping (Cu^2+^ ion-dopant of 1–5 mol%, concentration range of 50–200 mg mL^−1^) against *S. aureus* and *Escherichia coli*. Although high concentrations were needed in both cases to reduce bacterial growth, *E. coli* was inhibited more strongly.^49^ Conversely, Shu *et al.* (2020) showed greater growth reduction of *S. aureus* than of *E. coli* with Cu^2+^ ion-doped HAp.^50^ These contradictory results suggest strain-dependent mechanisms of copper protection or differences in antimicrobial activity based on nanoparticle synthesis methods.

No independent effect of orthosilicate group doping on the antibacterial properties of copper-doped nanohydroxyapatite was observed. Similarly, in our previous study,^36^ single-silicate group doping did not significantly affect the antibacterial properties of silver-doped nanosized hydroxyapatite compounds. However, a synergistic effect of dual doping on antifungal activity was observed. Unlike copper doping, which was effective only against Gram-positive bacteria, silver doping exhibited antibacterial activity against all five tested bacterial strains.

The antibacterial activity of Cu^2+^ ion-doped hydroxyapatite-type compounds enhances their potential for biomedical applications. Gram-positive bacteria are a major cause of nosocomial infections, including postoperative infections. Acute implant-related infections are often attributed to virulent strains of *Staphylococcus aureus*. However, *Staphylococcus epidermidis* and *Enterococcus faecalis* have also been reported as significant pathogens in post-surgical infections.^51–54^ The development of antimicrobial materials for implantology represents a promising strategy to combat such infections.^55^

**Fig. 8:**
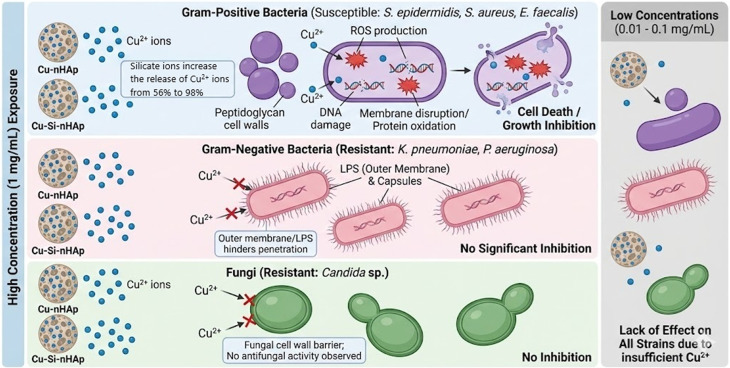
Predicted selective antibacterial mechanism of nHAp and Si-nHAp doped with Cu^2+^ ions.

The mechanisms underlying the antibacterial activity of apatite-type materials have been gradually elucidated through studies of their unique chemical structure, grain size, and metal-ion dopants. Apatite-type materials doped or co-doped with biologically active ions inhibit bacterial growth through several mechanisms, including inhibition of nucleic acid and protein synthesis, modification of bacterial cell membrane permeability, disruption of the cell membrane and cell wall, and inhibition of bacterial metabolism. In our case, the selective mechanism of action of Cu^2+^ ions is related to their release from the apatite-type structure (see Fig. 8).

### 3.6 Antifungal activity of nanosized particles

**Fig. 9:**
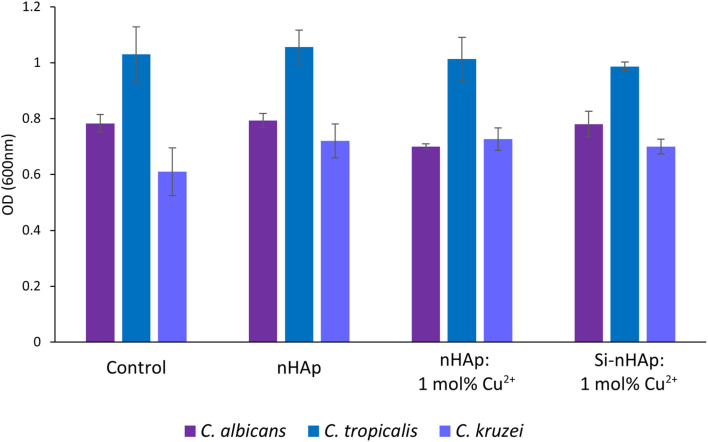
The inhibition of fungal (*C. albicans, C. kruzei*, and *C. tropicalis*) growth by nHAp,^36^ nHAp: 1 mol% Cu^2+^, and Si-nHAp: 1 mol% Cu^2+^ (concentration of 1 mg mL^−1^), fungi incubated without hydroxyapatite-type material were the control group (C). Mean ± SD, *n* = 3; *p < 0.05.

**Fig. 10:**
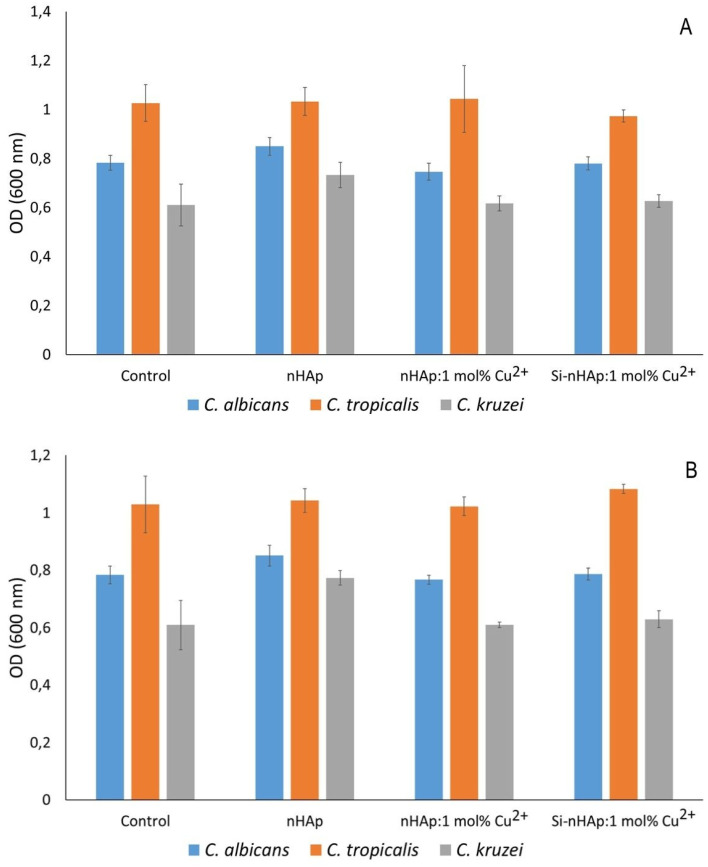
The effect of nHAp,^36^ nHAp: 1 mol% Cu^2+^, and Si-nHAp: 1 mol% Cu^2+^ ((A) – 0.01 mg mL; (B) – 0.1 mg mL^−1^) on fungal (*C. albicans*, *C. kruzei*, *and C. tropicalis*) growth; fungi incubated without hydroxyapatites were the control group. Mean ± SD, *n* = 3; *p < 0.05.

The results of the influence of Cu^2+^ ion-doped nHAps at 0.01, 0.1, and 1 mg mL^−1^ on the growth of *C. albicans*, *C. kruz*ei, and *C. tropicalis* are shown in Fig. 9 (1 mg mL^−1^) and Fig. 10 (0.01 and 0.1 mg mL^−1^). None of the tested hydroxyapatite-type materials exhibited antifungal activity, since there were no significant differences in growth compared to the control. Although the Cu^2+^ ion has documented antifungal activity against *Candida* sp*.* and filamentous fungi.^56,57^ Previous reports showed that the activity of Cu^2+^ ion-doped hydroxyapatite-type and fluorapatite-type materials against *C. albicans* was weak.^58^ On the other hand, Narayanan *et al.* (2020) reported high antimicrobial activity of hydroxyapatite-type materials containing Cu^2+^ ions, chitosan, and polyvinyl pyrrolidone against *C. albicans* and *Penicillium notatum*.^59^ This suggests that incorporating additional components into the nHAp structure may enhance its antifungal activity.

### 3.7 Cytocompatibility evaluation

**Fig. 11:**
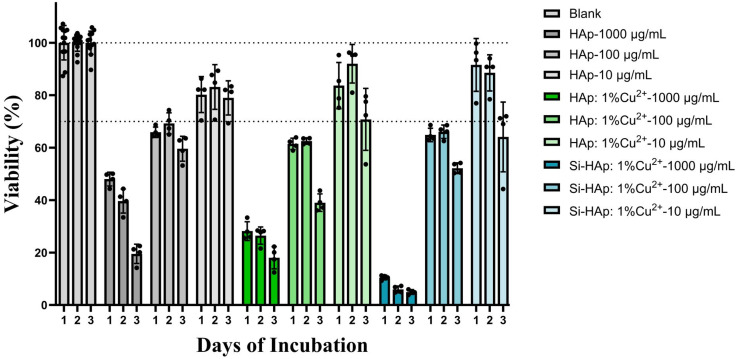
Cytocompatibility assessment of nHAp, nHAp: 1 mol% Cu^2+^, and Si-nHAp: 1 mol% Cu^2+^ against HUVECs as quantified by ATP content. Cells grown without hydroxyapatite materials were the blank group. Horizontal lines indicate 70% and 100% viability, corresponding to the ISO:10993–5 cytocompatibility cut-off and full viability, respectively. Mean ± SD; *n* ≥ 4.

The promising biocompatibility of nanosized hydroxyapatite materials has previously been described by our group and others.^16,60,61^ To investigate whether the inclusion of Cu^2+^ ions and orthosilicate groups alters this property, HUVECs were cultured in the presence of the nHAp over 72 hours (Fig. 11). At 0.01 µg mL^−1^, all groups exhibit fair cytocompatibility (over 70% viability) over the first two days, with a drop-off by day three for both Cu^2+^ ion-containing groups. At higher concentrations, cytotoxicity is observed, with HUVEC viability declining in a time- and dose-dependent manner. At bactericidal concentrations, cell viability in both Cu^2+^ ion-containing groups is very poor (≤30%). On days one and two, the unmodified nHAp at 1 mg mL^−1^ performs only slightly better at ∼40% viability, before similarly falling below 30% viability by day three. While this appears to indicate cytotoxicity due to the presence of the nanoparticles directly, this observation is likely a type of “false cytotoxicity” that is often seen *in vitro* for high specific surface area ceramic materials due to adsorption of ions, such as Ca^2+^, Mg^2+^, and HPO_4_^2−^ ions, from the cell culture media.^62^*In vivo*, such absorption is beneficial, as it can expedite integration with host bone and facilitate apatite formation. However, *in vitro*, where there is no continual fluid exchange, ion depletion occurs, leading to inhospitable conditions for cells. In our previous study, extractions from 100 mg mL^−1^ nHAp, 100× the highest concentration evaluated here, could be supplemented to replace the absorbed ions, resulting in significantly rescued cellular viability.^62^ Given this property of nHAp, it is more useful for our discussion to focus on comparisons to the performance of unmodified nHAp particles rather than absolute viability values. In this manner, we observe that all three nHAp groups perform comparably at 10 and 100 µg mL^−1^, whereas the Cu^2+^ ion-containing nHAps exhibit elevated toxicity at 1000 µg mL^−1^. This cytotoxicity is especially evident for the Si-doped group. These data suggest that at bactericidal concentrations, SiO_4_^4−^ group and Cu^2+^-ion-*co*-doped particles are potentially mildly cytotoxic to mammalian cells.

### 3.8 ICP-MS quantification of released ions

Release experiments were performed on samples at 1000 µg mL^−1^ and evaluated by ICP-MS. The results for the Cu^2+^ ions are summarized in Table 3. The raw data were calculated based on the total Cu^2+^ ion content in the materials, which was determined by ICP-OES (Table 2). As can be seen, more than 50% of Cu^2+^ ions are released from both the nHAp: 1 mol% Cu^2+^ and Si-nHAp: 1 mol% Cu^2+^ materials. Significantly more Cu^2+^ ions are released from the orthosilicate-doped samples (97.5%), suggesting silicon ions promote the release of Cu^2+^ ions from the apatite matrix. This enhanced Cu^2+^ leaching correlates with the cytocompatibility test (Fig. 10), where Cu^2+^ and SiO_4_^4−^ dual doping resulted in significantly decreased cell viability compared to Cu^2+^ doped nHAp. Previous literature suggests the Cu^2+^ ion-concentration necessary to begin eliciting cytotoxicity in HUVECs is ∼10 µM, which is far below 119.6 µM, the lowest release concentration observed here.^63^ The data also show that extending the release window beyond 24 hours does not lead to significantly higher amounts of leached Cu^2+^ ions entering the solution, suggesting an initial rapid release of available copper, which may create temporarily high local concentrations of Cu^2+^ ions. Together, the released Cu^2+^ ions appear to be responsible for the observed mild cytotoxicity of the Cu^2+^ ion-doped materials.

**Table 3:** Averaged release (µg mL^−1^) of Cu^2+^ ion from the tested materials incubated in cell-culture media, determined by the ICP-MS method. The percentage of released Cu^2+^ ions relative to the total Cu^2+^ ion content in the tested materials is based on the ICP-OES results from Table 2

Material	Cu^2+^ released [µg mL^−1^]	RSD [%]	Cu^2+^ released/Cu^2+^ content [%]
nHAp:1 mol% Cu^2+^, 24 h	7.6	0.7	47.7
nHAp:1 mol% Cu^2+^, 48 h	7.9	0.4	49.2
nHAp:1 mol% Cu^2+^, 72 h	9.0	0.1	56.1
Si-nHAp:1 mol% Cu^2+^, 24 h	12.5	1.1	81.6
Si-nHAp:1 mol% Cu^2+^, 48 h	14.9	1.2	97.5
Si-nHAp:1 mol% Cu^2+^, 72 h	12.7	0.5	83.1

## 4 Conclusions

This study demonstrated that combined cationic and anionic doping can effectively modify the morphology and antimicrobial activity of nanosized hydroxyapatite. The microwave-assisted hydrothermal synthesis followed by sintering at 450 °C enabled the preparation of nHAp: Cu^2+^ and dual-substituted Si-nHAp: Cu^2+^, containing low levels of dopants.

The low doping level of Cu^2+^ ions (1 mol%) and the presence of one orthosilicate group influence nanoparticle morphology and reduce its dimensions. Nanoparticle size tends to decrease following this order: nHAp > nHAp doped with Cu^2+^ > Si-nHAp doped with Cu^2+^.

The cytotoxicity assessment of the nanoparticles showed mild reductions in cell viability in the Cu^2+^-containing groups compared to nHAp, especially at higher concentrations, indicating Cu^2+^-mediated stress. The SiO_4_^4−^ nHAps exhibited greater cytotoxicity, consistent with the enhanced Cu^2+^ ion release rate observed by ICP-MS.

Both Cu^2+^ ion-doped and Cu^2+^ ion-doped orthosilicate-substituted hydroxyapatite nanoparticles exhibited selective antibacterial activity against Gram-positive bacteria strains (*Staphylococcus epidermidis*, *Staphylococcus aureus*, *Enterococcus faecalis*), while no significant activity was observed against Gram-negative bacteria (*Klebsiella pneumoniae*, *Pseudomonas aeruginosa*) and yeast species (*Candida albicans*, *Candida kruzei*, *Candida tropicalis*).

These results suggest strain-specific activity of the doped nanosized hydroxyapatite and demonstrate their potential as platforms for designing biomaterials with selective antibacterial properties and controlled ion release to prevent infection caused by Gram-positive bacteria.

## Author contributions

Nataliia D. Pinchuk: conceptualization, methodology, investigation, writing – original draft, writing – review & editing, visualization, formal analysis, data curation, project administration. Agata Piecuch: methodology, investigation, writing – original draft, writing – review & editing, visualization, formal analysis, data curation. Paulina Sobierajska: conceptualization, methodology, investigation, writing – original draft, writing – review & editing, visualization, formal analysis, data curation, project administration, validation. Cole Latvis: methodology, investigation, visualization, writing – original draft, data curation, writing – review & editing. Szyszka Katarzyna: methodology, formal analysis, visualization, writing – review & editing. Sara Targońska: methodology, investigation, writing – review & editing. Oleksii Bezkrovnyi: methodology, investigation. Rafał Ogórek: formal analysis, resources. Yadong Wang: resources, writing – review & editing. Rafal J. Wiglusz: supervision, conceptualization, methodology, investigation, resources, funding acquisition, formal analysis, data curation, visualization, writing – original draft, writing – review & editing, project administration.

## Conflicts of interest

There are no conflicts to declare.

## Data Availability

The authors declare that data will be made available by the corresponding author upon reasonable request. All data generated or analyzed during this study have been included in the article entitled “Synergistic effect of copper(ii) ions and orthosilicate groups in nanosized hydroxyapatite.
